# Neuroimaging in vascular cognitive impairment: a state-of-the-art review

**DOI:** 10.1186/s12916-016-0725-0

**Published:** 2016-11-03

**Authors:** Wolf-Dieter Heiss, Gary A. Rosenberg, Alexander Thiel, Rok Berlot, Jacques de Reuck

**Affiliations:** 1 Max Planck Institute for Metabolism Research, Gleueler str. 50, D-50931 Cologne, Germany; 2 Department of Neurology, UNM Memory and Aging Center, MSC 11 6035, University of New Mexico, Albuquerque, NM 87131 USA; 3 Department of Neurology & Neurosurgery, McGill University at SMBD Jewish General Hospital and Lady Davis Institute for Medical Research, Montreal, H3T 1E2 Québec Canada; 4 Department of Neurology (R.B.), University Medical Centre Ljubljana, 1000 Ljubljana, Slovenia; 5 INSERM U1171, Degenerative and Vascular Cognitive Disorders, Université Lille 2, Lille, France

**Keywords:** Neuroimaging, CT, MRI, PET, Vascular cognitive impairment, Cerebral small vessel disease, Molecular imaging

## Abstract

Imaging is critical in the diagnosis and treatment of dementia, particularly in vascular cognitive impairment, due to the visualization of ischemic and hemorrhagic injury of gray and white matter. Magnetic resonance imaging (MRI) and positron emission tomography (PET) provide structural and functional information. Clinical MRI is both generally available and versatile – T2-weighted images show infarcts, FLAIR shows white matter changes and lacunar infarcts, and susceptibility-weighted images reveal microbleeds. Diffusion MRI adds another dimension by showing graded damage to white matter, making it more sensitive to white matter injury than FLAIR. Regions of neuroinflammatory disruption of the blood–brain barrier with increased permeability can be quantified and visualized with dynamic contrast-enhanced MRI. PET shows metabolism of glucose and accumulation of amyloid and tau, which is useful in showing abnormal metabolism in Alzheimer’s disease. Combining MRI and PET allows identification of patients with mixed dementia, with MRI showing white matter injury and PET demonstrating regional impairment of glucose metabolism and deposition of amyloid. Excellent anatomical detail can be observed with 7.0-Tesla MRI. Imaging is the optimal method to follow the effect of treatments since changes in MRI scans are seen prior to those in cognition. This review describes the role of various imaging modalities in the diagnosis and treatment of vascular cognitive impairment.

## Background

Vascular etiologies are among the most common causes of dementia, but the numbers vary considerably according to the different criteria used for vascular cognitive impairment (VCI) [1, 2]. According to a controlled neuropathological study, pure vascular disease is responsible for 8–10 %, Alzheimer’s disease (AD) for 60–70 %, and dementia with Lewy bodies (DLB) for 10–25 % of dementia cases [3]. In the Rochester Epidemiology Project of 419 old demented patients, the post mortem diagnosis of AD was established in 51 %, of pure vascular dementia in 13 %, and of mixed vascular-Alzheimer dementia in 12 % of patients, with “other” diagnosis in the remaining patients [4].

Furthermore, it is evident from autopsy studies that many patients with mixed dementia have both vascular and degenerative causes [3, 5]. The heterogeneity of patients included in the VCI diagnosis has resulted in attempts to refine the definitions and identify subgroups of patients [6]. The three main causes of VCI are large vessel strokes (macroangiopathy, arteriosclerosis), small vessel disease (SVD; microangiopathy, arteriolosclerosis), and microhemorrhages. Large vessel disease may cause thrombosis or embolus with or without involvement of white matter [7]. SVD causes incomplete or complete infarcts, lacunar infarcts in both white matter and subcortical gray matter nuclei, and diffuse injury in white matter [8]. A characteristic feature of SVD is the sparing of U-fibers that connect adjacent regions of the cortex. The growth of white matter hyperintensities (WMHs) occurs gradually over many years. Large population studies have reported that FLAIR imaging shows changes in white matter [9]; however, many elderly people over the age of 65 show white matter changes on magnetic resonance imaging (MRI) that do not necessarily correspond with symptoms [10]. These changes in white matter are referred to as leukoaraiosis, which is a non-specific term to indicate rarefied white matter for which the underlying pathology is not known, and it should not be used to imply a symptom-producing lesion [11]. Since clinical signs and symptoms are often insufficient to allow for a final diagnosis and usually cannot differentiate among the various etiologies, neuroimaging plays an important role in the management of patients with impaired cognition.

## Methods

For this review, based on the lectures presented at the International Congress of Vascular Dementia in Ljubljana 2015, additional literature was searched in PubMed and relevant publications were selected with special prioritization to publications published over the last 10 years. Due to limitations in space, a complete coverage of the extensive literature on this widespread topic was not possible.

### Correlating neuroimaging with morphologic substrates

Neuroimaging provides important information on the neuroanatomical substrate of the disorder, plays an important role in the diagnosis, and adds to the prediction of VCI. Most acute stroke patients undergo brain imaging by computed tomography (CT); thus, studies using CT are representative of the whole clinical population. In clinical practice, CT is performed primarily to exclude hemorrhage and stroke mimics (such as brain tumors), and can often demonstrate early signs of ischemia (e.g., swelling, hypodensity, and hyperdense vessels) and old stroke lesions. Furthermore, the presence and severity of WMHs and brain atrophy can also be readily determined from CT brain scans, which may predict subsequent cognitive impairment and dementia. There is good agreement between brain atrophy and moderate to severe white matter lesions on CT and MRI measurements [12, 13].

MRI remains the key neuroimaging modality in VCI (review in [14]). Unless contraindicated, MRI is preferred to CT for research and routine clinical use due to its higher sensitivity and specificity for detecting pathological changes [15]. Standards for neuroimaging with a widely accepted terminology permitting comparison of findings between centers have been recommended (STandards for ReportIng Vascular changes on nEuroimaging, STRIVE) [16]. Numerous studies identified MRI markers of SVD (lacunes, WMHs, cerebral microbleeds, silent infarcts, cerebral atrophy) as determinants of VCI. Vascular lesions traditionally attributed to VCI comprise subcortical areas of the brain, especially subfrontal white matter circuits, strategic areas of single infarction such as the dominant thalamus or angular gyrus, deep frontal areas and the left hemisphere, and bilateral brain infarcts or volume-driven cortical-subcortical infarctions reaching a critical threshold of tissue loss or injury [17]. Multiple punctuate or confluent lesions can be seen in white matter by MRI and are termed leukoaraiosis [11], which is a non-specific term; these changes are often seen in healthy elderly subjects and in subjects with migraine. SVD often causes incomplete infarcts. Extended subcortical SVD may be associated with the pathology of Binswanger’s disease [18]. Some studies have suggested a threshold of 10 cm^2^ [19] or 25 % of total white matter [20] as the lesion load to affect cognition. Incomplete infarcts present as hyperintensities on FLAIR images, whereas complete infarcts present as hypointense lesions in relation to the brain and isointense to the cerebrospinal fluid.

The third major neuroimaging aspect of VCI are microhemorrhages, and were found in up to 65 % of VCI cases [21]. Macrohemorrhages associated with cognitive impairment (e.g., venous infarcts) can be seen on conventional T1- and T2-weighted spin echo images, microhemorrhages can be detected accurately using T2*-weighted gradient echo images [22]. Microhemorrhages and white matter changes not only occur in vascular dementia but also in neurodegenerative diseases [23, 24]. In addition, recent papers have suggested that only minor percentages of WMHs on MRI are explained by hypertension [25], and even microbleeds can be caused by other novel factors such as infection [26].

### High resolution MRI for neuropathological investigation of vascular dementia syndromes

7.0-Tesla (T) MRI can be used as an additional tool to examine post mortem brains of patients with neurodegenerative and vascular dementia syndromes [27]. High-field MRI shows the degree and the distribution of the cerebral atrophy, and it detects lesions that can be selected for histological examination. Small cerebrovascular lesions can be quantified and the iron load evaluated.

High-resolution 7.0-T MRI allows detection of cortical microinfarcts in vivo [28]. There is some evidence that cortical microinfarcts can be visualized in vivo at a 3.0-T field strength using newer sequences such as double inversion recovery [29]. However, this finding has not been verified histopathologically. A recent study has found some supportive evidence for use of double-inversion recovery sequence as a marker of cortical ischemic lesions based on the relationship with carotid atherosclerosis [30]. Further studies with a focus on clinicopathological correlation are required before these sequences will find their way into clinical practice.

Detection of small cortical bleeds has a reliability of 96 % [31] since cortical microbleeds predominate to a different degree in the frontal areas of all neurodegenerative disease groups compared to the controls [32]. Cortical microinfarcts are more frequent in vascular dementia, in Lewy body, and in AD associated with severe cerebral amyloid angiopathy [33]. Cerebellar microinfarcts, on the other hand, are mainly due to atherosclerotic disease [34].

Lacunes and white matter changes are mainly observed in vascular dementia brains. The latter are also frequently seen in frontotemporal lobar degeneration due to Wallerian degeneration, rather than caused by cerebrovascular disease [35]. Superficial siderosis is due to hemosiderin deposition in the subpial layer and is associated with an underlying cortical lesion, which can be either a hemorrhage or an infarct after hemorrhagic transformation [36]. Iron deposition in the basal ganglia is significantly increased in frontotemporal lobar degeneration [37].

### Diffusion MRI in SVD and cognitive decline

Cognitive decline in healthy ageing and age-related disorders is related to cortical “disconnection” – white matter damage leading to reduced functional integration among distant cortical areas [38]. In particular, white matter alterations are the feature of subcortical ischemic vascular disease (SIVD) that helps establish a diagnosis. However, there is only limited correlation in SIVD between cognition and the extent of white matter alterations observed by standard MRI sequences such as FLAIR [39]. In contrast, diffusion-weighted imaging can characterize microstructural alterations in the normal-appearing white matter, which is also affected in VCI due to local pathology as well as Wallerian degeneration from distant lesions. Diffusion MRI indices of normal-appearing white matter exhibit a higher correlation with cognition than conventional MRI markers [40]. Another advantage of the use of diffusion MRI in SIVD is that it provides quantitative markers of tissue integrity [41].

The contrast provided by diffusion MRI is based on the thermal motion of water molecules, which is hindered by cellular membranes and myelin sheaths [42]. In most cases, diffusion is anisotropic in organized white matter structures, where it exhibits directional preponderance. On the other hand, diffusion is isotropic in areas where there is less microstructural organization of white matter fibers such as grey matter or cerebrospinal fluid [43]. Several indices of white matter integrity can be calculated. Mean diffusivity quantifies the extent of total diffusivity in a given voxel, whilst axial and radial diffusivity quantify the amount of diffusion along and perpendicular to its main direction, respectively. Another commonly applied metric is that of fractional anisotropy, which is a relative measure that quantifies the amount of directional preponderance of diffusion in a given voxel. Diffusion MRI indices are changing both with healthy ageing and disease. Cellular damage leads, in general, to less restricted diffusion and, in turn, to an increase in absolute diffusivity and decreased anisotropy. Fractional anisotropy is highly sensitive to microstructural changes, but is not very specific to the type of change. Mean, axial, and radial diffusivity provide complementary information on the nature of microstructural alterations. However, there is no simple relationship between individual metrics and white matter integrity, and it is advantageous that several metrics are used to provide a fuller characterization of microstructural alterations [44, 45]. These can be assessed at various spatial scales.

Histogram distributions of diffusion metrics provide useful markers of the disease process that are sensitive to change [46] and correlate well with clinical progression [47, 48]. In CADASIL, a mean diffusivity value has been identified as the main predictor of clinical progression among other demographic, clinical, and conventional MRI markers for various clinical endpoints, including disability, cognition, and newly occurring strokes [48]. Histogram measures are highly reproducible and thus provide robust summary statistics in SIVD [49]. One of their limitations is that they do not provide information solely on intrinsic microstructural changes, but are also influenced by volumetric alterations, which can introduce bias in populations prone to atrophy, but can be corrected using post-processing techniques [50]. In addition to global summary measures, diffusion MRI can be used to estimate the spatial profile of white matter alterations. Voxel-wise analyses, as well as tractography studies, have demonstrated critical areas within the damaged white matter that correlate most strongly with aspects of cognition such as executive function or verbal memory [51–53]. Estimating white matter alterations in individual regions or single tracts can explain the profile of cognitive impairment in a given patient with VCI as well as helping to understand the relative importance of a small number of strategically-located lesions versus the cumulative effect of multiple lesions; both could prove fruitful longitudinal studies on disease progression as well as intervention studies.

Whole-brain tractograms can be used to reconstruct white matter structural networks. Their topology measures are quantified using graph theory-based metrics such as measures of network integration. Networks of patients with SIVD and cerebral amyloid angiopathy exhibit a less efficient topology associated with cognitive decline [54, 55]. Network metrics have been shown to partly or fully explain the association between cognition and other MRI measures commonly used in SIVD, including mean fractional anisotropy and mean diffusivity [54]. Thus, network metrics provide useful markers of the disease process as well as suggesting the importance of network disruption as a potential common mechanism of how different types of vascular damage can lead to cognitive decline.

Diffusion MRI allows assessment of subtle alterations in SIVD that are not captured by other imaging techniques and provides several markers of micro- and macrostructural organization that are sensitive to change and related to important clinical endpoints. Linking different levels of spatial analysis remains an important challenge in understanding the pathophysiology of SIVD and cognitive decline. Importantly, it also has the potential to predict cognitive trajectories in individual patients as well as to help establish a diagnosis; however, further studies are required in these areas.

### Proton MR spectroscopy and dynamic contrast-enhanced MRI

Detection of ischemic changes in white matter as opposed to those due to aging is possible with proton magnetic resonance spectroscopy (^1^H-MRS), diffusion tensor imaging, and dynamic contrast-enhanced MRI (DCEMRI). ^1^H-MRS shows injury to the axons by measuring the levels of N-acetylaspartate and creatine [56, 57]. Diffusion tensor imaging provides another indicator of structural damage to white matter [58]. Finally, DCEMRI is a functional measure of the leakiness of blood vessels, which indicates the presence of neuroinflammation [59, 60]. The combination of multiple modalities provides a clear picture of the extent of damage and the possible etiology of the injury in white matter. Using these modalities, white matter changes due to ageing can be separated from structural and functional changes due to pathology.

An important aspect of the pathological changes seen in the small vessel type of VCI is the measurement of blood–brain barrier (BBB) permeability. Quantitative regional measurements of BBB can be made with DCEMRI, which requires the injection of MRI contrast agents [59]. They also have high computational needs and remain unstandardized so that values from different sites are difficult to compare.

There is general agreement that the small vessel form of VCI, which generally causes progressive damage to white matter, is the optimal form for treatment trials. The major challenges facing the next stage of VCI imaging research are (1) the identification of imaging patterns characteristic of Binswanger’s disease, and (2) the selection of the imaging modalities that undergo changes over time and which could be used as surrogate markers for treatment trials [61].

### Molecular imaging in the differential diagnosis of vascular dementia

**Fig. 1:**
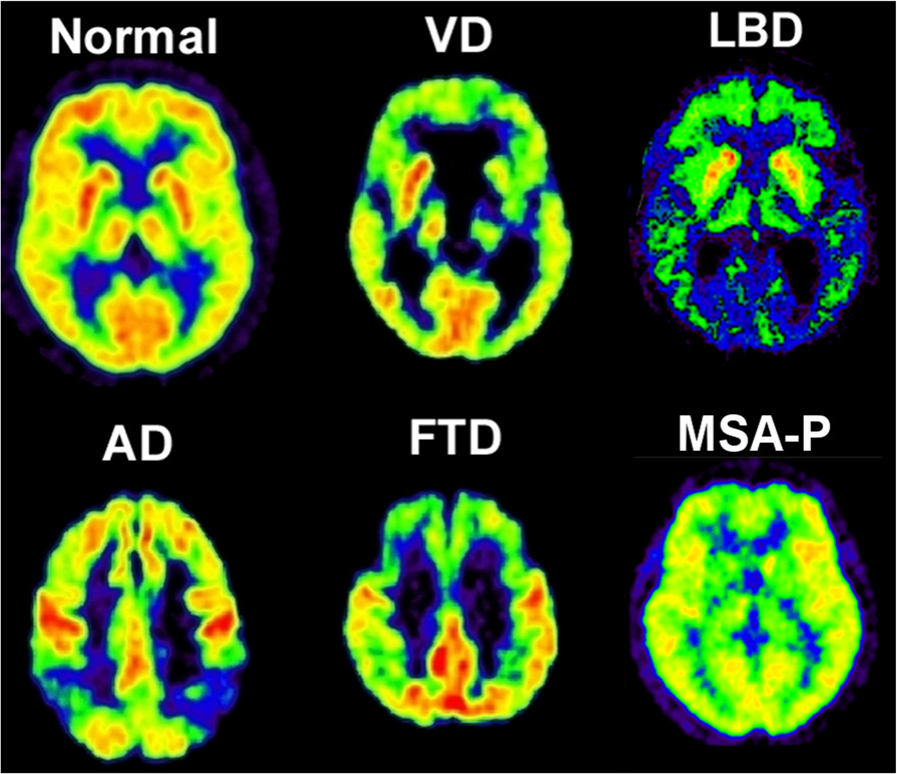
Typical metabolic patterns for different types of dementia compared to normal controls and vascular dementia (VD). Alzheimer’s disease (AD), frontotemporal dementia (FTD), and Lewy-Body dementia (LBD) show distinct cortical patterns of decreased metabolism, while multisystem atrophy type P (MSD-P) shows a decreased metabolism in the putamen on both sides. In contrast, a typical feature of VD is the simultaneous occurrence of patchy, often asymmetrical cortical and subcortical areas of decreased glucose metabolism

Positron emission tomography (PET) can support the clinical diagnosis by visualizing cerebral functions in typically affected brain regions. PET of ^18^F-2-fluoro-2-deoxy-D-glucose (FDG) for measurement of regional cerebral glucose metabolism (rCMRglc) has shown a typical metabolic pattern in patients with probable AD: hypometabolism in temporoparietal and frontal association areas, but relative recessing of primary cortical areas, basal ganglia, and cerebellum (Fig. 1). In VCI, a different pattern is seen (review in [62]), where FDG-PET detects regions of focal cortical and subcortical hypometabolism, a metabolic pattern different from that typical for AD with marked hypometabolism affecting the association areas [63]. A significant reduction of rCMRglc was observed in widespread cerebral regions (middle frontal cortex, temporoparietal cortex, basal ganglia, cerebellum, and brainstem) [64]. Hypometabolism was more marked in subcortical areas and primary sensorimotor cortex and the association areas were less affected than in AD. A metabolic ratio (rCMRglc of association areas divided by rCMRglc of primary areas, basal ganglia, cerebellum, and brainstem) was significantly lower in AD than in VCI. A single region that could discriminate between VCI and AD could not be identified, but small infarcts, in combination with WMHs, may contribute to cognitive decline. Rather than the total volume of infarction, data from PET studies indicate that the volume of functional tissue loss is more important than the extent of morphological lesions, since this includes incompletely infarcted tissue and morphologically intact but deafferented cortex.

The accuracy of rCMRglc changes for the clinical diagnosis of AD has only been investigated in few reports. An analysis of receiver operating characteristics recorded 93 % sensitivity and 83 % specificity for differentiation of patients with probable AD from those without AD or other dementing illnesses [65]. A significantly abnormal metabolic ratio in subjects with mild cognitive impairment (MCI) indicated a high risk to develop dementia within the next 2 years. SIVD could be distinguished from clinically probable AD by a more diffuse pattern of hypometabolism involving also the primary cortices, basal ganglia, thalamus, and cerebellum.

Characteristic patterns of regional hypometabolism are also seen in other degenerative dementias (review in [66]) (Fig. 1), such as frontotemporal dementia, clinically conspicuous by changes in personality and behavior, semantic deficits, and progressive aphasia associated with distinct often asymmetric frontal or frontotemporal metabolic changes that are typically centered in the frontolateral cortex and the anterior pole of the temporal lobe. DLB, namely fluctuating consciousness, Parkinsonian symptoms, and impairment of visual perception including hallucinations, is characterized by a reduction of glucose metabolism in the primary visual cortex in addition to that in posterior association areas. Other degenerative disorders show typical hypometabolism in the specifically affected brain structures: the putamen and cortex in corticobasal degeneration, the caudate nucleus in Huntington’s disease, the frontal cortex and midbrain in progressive supranuclear palsy, and pons and cerebellum in olivopontocerebellar atrophy. Depressive disorders may mimic cognitive impairment; in these cases, glucose metabolism does not show regional abnormalities typical for the degenerative disorders [67].

### Imaging synaptic transmission and accumulation of pathologic proteins

**Fig. 2:**
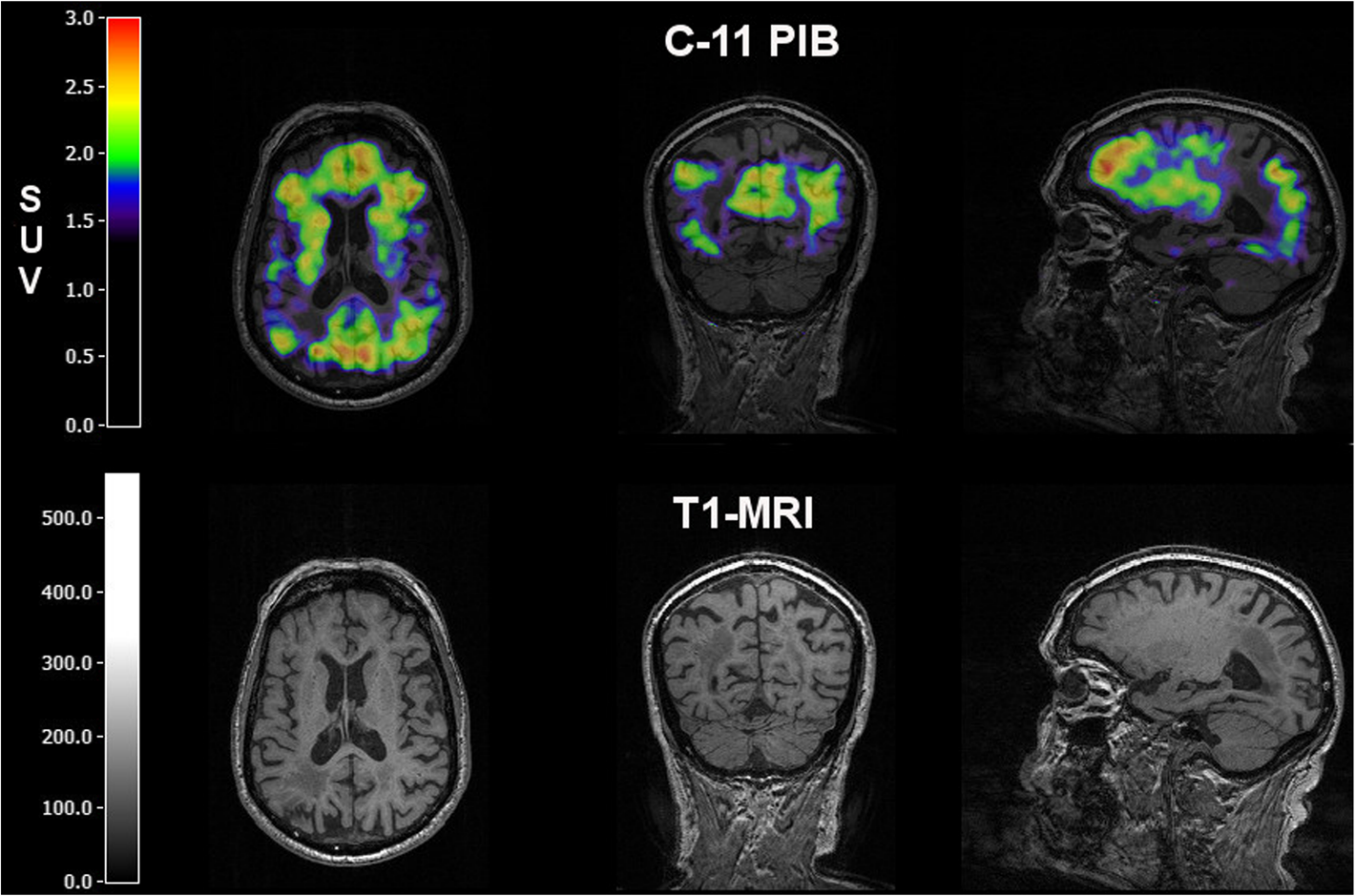
An 82-year-old man with cognitive decline 6 months after right parietal ischemic stroke. 11-C-PIB-PET shows amyloid deposits in brain regions typical for Alzheimer’s disease, thus differentiating Alzheimer’s dementia from post-stroke dementia as a possible differential diagnosis

Additional PET tracers can further support the diagnosis of a type of dementia and also yield information on the underlying pathophysiology. Tracers permit the study of selectively affected transmitter/receptor systems, e.g., the cholinergic system in AD, where a significant reduction of cholinergic activity in the cortex of AD patients and those with MCI and early conversion to AD is observed [68], or the dopaminergic system in DLB [69] and the detection of pathogenetic depositions, e.g., amyloid and tau in AD [70] or inflammatory reactions with microglia activations as in VCI. In particular, the imaging of accumulation of pathologic proteins is a recent strategy to differentiate degenerative dementias. Amyloid is a pathogenetic product in the development of AD and its accumulation is a key finding in this disease (Fig. 2) and can be imaged by ^11^C-labeled Pittsburgh Compound B (PiB) [71] or by several newer ^18^F-labeled tracers [72]. Whereas only small amounts of amyloid can be detected in white matter in normal aging [73], accumulation is visible in the frontal and temporoparietal cortex in AD and MCI. However, in 20–30 % of aged persons without relevant cognitive impairment, an increased accumulation of amyloid can also be detected [74], and the grade of amyloid deposition as detected by PET is not related to the severity of cognitive impairment [75]. Therefore, amyloid might be deposited in the brain long before cognitive impairment is recognized.

A more specific pathologic protein produced in AD is tau, and its deposition in the mesial temporal lobe is an early marker of AD or MCI [76], with the amount of tau detected in the cortex by selective PET-tracers being related to the severity of cognitive impairment [77]. These PET-tracers also detect the primary pathological substrate in other degenerative dementias (e.g., tau in frontotemporal dementia) [78] and permit the differentiation between AD and VCI and other degenerative dementias. As these studies provide insight on the early changes of these diseases, selective PET-studies might be useful to detect preclinical stages in which therapeutic efforts might be promising.

### PET and imaging of neuroinflammation

Using amyloid imaging tracers such as ^11^C-PiB, it has been shown that patients with radiologically defined SIVD and amyloid deposits were, on average, older, had worse cognitive performance, fewer lacunar infarcts, and more hippocampal atrophy than amyloid-negative patients. Amyloid deposits were observed in approximately 30 % of patients with radiologically-defined VCI. These findings may indicate a synergistic effect of amyloid depositions and vascular lesions since both are known risk factors for developing dementia [79, 80]. In analogy to these clinical studies, animal models suggest that it may be stroke-induced inflammation (rather than the ischemic event itself) which acts synergistically with amyloid depositions and accelerates cognitive decline [81]. Direct proof for this relationship, however, remains to be established in human stroke.

Several PET tracers (such as ^11^C-[R]-PK11195 and others) have been developed to measure the activity of microglia (the most important cellular marker of neuroinflammation) in the ischemic brain in vivo and can be used to address these questions [82]. While a direct relationship between cortical microglia activity and cognitive performance in dementia remains to be demonstrated [6, 83], it is known, from post mortem [84] and in vivo imaging studies [85], that ischemia-induced neuroinflammation can trigger ongoing neurodegenerative processes of fiber tracts. This inflammation-associated tract degeneration does not only directly affect neurons that were subject to ischemia but can spread trans-synaptically [86] and thus compromise larger scale networks. In a pilot study, two PET-scans were performed 5–7 months following an ischemic stroke to assess amyloid deposition (^11^C-PiB) and microglia activation (^11^C-[R]-PK11195). Cognitive performance 5–7 months after the stroke was negatively correlated with gray matter amyloid deposition and this relationship remained significant even when initial cognitive performance and age were entered as covariates into the analysis. Similarly, microglia activation in the stroke-affected hemisphere white matter was highly correlated with cognitive performance [87]. The results of this study in human stroke may suggest that cortical amyloid deposition and post-stroke white matter inflammation contribute to post-stroke cognitive impairment and may constitute separate pathomechanisms to explain cognitive decline. If confirmed in larger trials, this finding might offer possibilities for clinical intervention to prevent post-stroke cognitive decline by modulation of inflammation or amyloid deposition.

## Conclusions

Neuroimaging will continue to play a leading role in the diagnosis of patients with dementia. While MRI is the most widely used modality and is available in most centers, PET offers the ability to distinguish between the vascular and neurodegenerative causes of dementia. Further, ^1^H-MRS, diffusion tensor imaging, and DCEMRI augment clinical MRI studies by showing ischemic damage to white matter and disruption of the BBB, a major factor in neuroinflammation. It is important to separate patients with mainly AD from those with mainly VCI; however, in reality, the majority of patients appear to have combinations of both. Planning clinical trials of patients with VCI is a critical need, and the ability to more clearly delineate between AD, VCI, and mixed pathology will be crucial to reduce the number of patients needed for a trial. A longitudinal study comparing the development of clinical symptoms with changes in imaging, including various MRI parameters and eventually quantitative data from PET followed by validation through neuropathological confirmation, would be the ideal basis for long-term treatment studies.

## References

[1] Rockwood K, Wentzel C, Hachinski V, Hogan DB, MacKnight C, McDowell I (2000). Prevalence and outcomes of vascular cognitive impairment. Vascular Cognitive Impairment Investigators of the Canadian Study of Health and Aging. Neurology.

[2] De Reuck J, Deramecourt V, Cordonnier C, Pasquier F, Leys D, Maurage CA, Bordet R (2016). The incidence of post-mortem neurodegenerative and cerebrovascular pathology in mixed dementia. J Neurol Sci.

[3] Jellinger KA (2007). The enigma of vascular cognitive disorder and vascular dementia. Acta Neuropathol.

[4] Knopman DS, Parisi JE, Boeve BF, Cha RH, Apaydin H, Salviati A, Edland SD, Rocca WA (2003). Vascular dementia in a population-based autopsy study. Arch Neurol.

[5] Korczyn AD (2002). Mixed dementia--the most common cause of dementia. Ann N Y Acad Sci.

[6] Rosenberg GA, Bjerke M, Wallin A (2014). Multimodal markers of inflammation in the subcortical ischemic vascular disease type of vascular cognitive impairment. Stroke.

[7] Gorelick PB, Scuteri A, Black SE, Decarli C, Greenberg SM, Iadecola C, Launer LJ, Laurent S, Lopez OL, Nyenhuis D (2011). Vascular contributions to cognitive impairment and dementia: a statement for healthcare professionals from the American Heart Association/American Stroke Association. Stroke.

[8] Roman GC, Erkinjuntti T, Wallin A, Pantoni L, Chui HC (2002). Subcortical ischaemic vascular dementia. Lancet Neurol.

[9] Vermeer SE, Longstreth WT, Koudstaal PJ (2007). Silent brain infarcts: a systematic review. Lancet Neurol.

[10] Hunt AL, Orrison WW, Yeo RA, Haaland KY, Rhyne RL, Garry PJ, Rosenberg GA (1989). Clinical significance of MRI white matter lesions in the elderly. Neurology.

[11] Hachinski VC, Potter P, Merskey H (1987). Leuko-araiosis. Arch Neurol.

[12] Wattjes MP, Henneman WJ, van der Flier WM, de Vries O, Traber F, Geurts JJ, Scheltens P, Vrenken H, Barkhof F (2009). Diagnostic imaging of patients in a memory clinic: comparison of MR imaging and 64-detector row CT. Radiology.

[13] Wahlund LO, Barkhof F, Fazekas F, Bronge L, Augustin M, Sjogren M, Wallin A, Ader H, Leys D, Pantoni L (2001). A new rating scale for age-related white matter changes applicable to MRI and CT. Stroke.

[14] Vitali P, Migliaccio R, Agosta F, Rosen HJ, Geschwind MD (2008). Neuroimaging in dementia. Semin Neurol.

[15] Brainin M, Tuomilehto J, Heiss WD, Bornstein NM, Bath PM, Teuschl Y, Richard E, Guekht A, Quinn T (2015). Post stroke cognition study group. Post-stroke cognitive decline: an update and perspectives for clinical research. Eur J Neurol.

[16] Wardlaw JM, Smith EE, Biessels GJ, Cordonnier C, Fazekas F, Frayne R, Lindley RI, O’Brien JT, Barkhof F, Benavente OR (2013). Neuroimaging standards for research into small vessel disease and its contribution to ageing and neurodegeneration. Lancet Neurol.

[17] Grysiewicz R, Gorelick PB (2012). Key neuroanatomical structures for post-stroke cognitive impairment. Curr Neurol Neurosci Rep.

[18] Roman GC (1987). Senile dementia of the Binswanger type. A vascular form of dementia in the elderly. JAMA.

[19] Boone KB, Miller BL, Lesser IM, Mehringer CM, Hill Gutierrez E, Goldberg MA, Berman NG (1992). Neuropsychological correlates of white-matter lesions in healthy elderly subjects. A threshold effect. Arch Neurol.

[20] van Straaten EC, Scheltens P, Knol DL, van Buchem MA, van Dijk EJ, Hofman PA, Karas G, Kjartansson O, de Leeuw FE, Prins ND (2003). Operational definitions for the NINDS-AIREN criteria for vascular dementia: an interobserver study. Stroke.

[21] Cordonnier C, van der Flier WM, Sluimer JD, Leys D, Barkhof F, Scheltens P (2006). Prevalence and severity of microbleeds in a memory clinic setting. Neurology.

[22] Koennecke HC (2006). Cerebral microbleeds on MRI: prevalence, associations, and potential clinical implications. Neurology.

[23] De Reuck J, Deramecourt V, Cordonnier C, Leys D, Pasquier F, Maurage CA (2011). Prevalence of small cerebral bleeds in patients with a neurodegenerative dementia: a neuropathological study. J Neurol Sci.

[24] De Reuck JL, Auger F, Durieux N, Cordonnier C, Deramecourt V, Pasquier F, Maurage CA, Leys D, Bordet R (2016). The topography of cortical microinfarcts in neurodegenerative diseases and in vascular dementia: a postmortem 7.0-Tesla magnetic resonance imaging study. Eur Neurol.

[25] Wardlaw JM, Allerhand M, Doubal FN, Valdes Hernandez M, Morris Z, Gow AJ, Bastin M, Starr JM, Dennis MS, Deary IJ (2014). Vascular risk factors, large-artery atheroma, and brain white matter hyperintensities. Neurology.

[26] Ihara M, Yamamoto Y (2016). Emerging evidence for pathogenesis of sporadic cerebral small vessel disease. Stroke.

[27] Wardlaw JM (2011). Post-mortem MR, brain imaging comparison with macro- and histopathology: useful, important and underused. Cerebrovasc Dis.

[28] van Veluw SJ, Zwanenburg JJ, Engelen-Lee J, Spliet WG, Hendrikse J, Luijten PR, Biessels GJ (2013). In vivo detection of cerebral cortical microinfarcts with high-resolution 7 T MRI. J Cereb Blood Flow Metab.

[29] Ii Y, Maeda M, Kida H, Matsuo K, Shindo A, Taniguchi A, Tomimoto H (2013). In vivo detection of cortical microinfarcts on ultrahigh-field MRI. J Neuroimaging.

[30] Landi D, Maggio P, Lupoi D, Palazzo P, Altamura C, Falato E, Altavilla R, Vollaro S, Coniglio AD, Tibuzzi F (2015). Cortical ischemic lesion burden measured by DIR is related to carotid artery disease severity. Cerebrovasc Dis.

[31] De Reuck J, Auger F, Cordonnier C, Deramecourt V, Durieux N, Pasquier F, Bordet R, Maurage CA, Leys D (2011). Comparison of 7.0-T T*-magnetic resonance imaging of cerebral bleeds in post-mortem brain sections of Alzheimer patients with their neuropathological correlates. Cerebrovasc Dis.

[32] De Reuck J, Auger F, Durieux N, Deramecourt V, Cordonnier C, Pasquier F, Maurage CA, Leys D, Bordet R (2015). Topography of cortical microbleeds in Alzheimer’s disease with and without cerebral amyloid angiopathy: a post-mortem 7.0-Tesla MRI study. Aging Dis.

[33] De Reuck J, Deramecourt V, Auger F, Durieux N, Cordonnier C, Devos D, Defebvre L, Moreau C, Caparros-Lefebvre D, Bordet R (2014). Post-mortem 7.0-tesla magnetic resonance study of cortical microinfarcts in neurodegenerative diseases and vascular dementia with neuropathological correlates. J Neurol Sci.

[34] De Reuck JL, Deramecourt V, Auger F, Durieux N, Cordonnier C, Devos D, Defebvre L, Moreau C, Capparos-Lefebvre D, Pasquier F (2015). The significance of cortical cerebellar microbleeds and microinfarcts in neurodegenerative and cerebrovascular diseases. A post-mortem 7.0-tesla magnetic resonance study with neuropathological correlates. Cerebrovasc Dis.

[35] De Reuck J, Deramecourt V, Cordonnier C, Auger F, Durieux N, Bordet R, Maurage CA, Leys D, Pasquier F (2012). Detection of microbleeds in post-mortem brains of patients with frontotemporal lobar degeneration: a 7.0-Tesla magnetic resonance imaging study with neuropathological correlates. Eur J Neurol.

[36] De Reuck J, Deramecourt V, Cordonnier C, Auger F, Durieux N, Pasquier F, Bordet R, Defebvre L, Caparros-Lefebvre D, Maurage CA (2013). Superficial siderosis of the central nervous system: a post-mortem 7.0-tesla magnetic resonance imaging study with neuropathological correlates. Cerebrovasc Dis.

[37] De Reuck JL, Deramecourt V, Auger F, Durieux N, Cordonnier C, Devos D, Defebvre L, Moreau C, Caparros-Lefebvre D, Leys D (2014). Iron deposits in post-mortem brains of patients with neurodegenerative and cerebrovascular diseases: a semi-quantitative 7.0 T magnetic resonance imaging study. Eur J Neurol.

[38] O’Sullivan M, Jones DK, Summers PE, Morris RG, Williams SC, Markus HS (2001). Evidence for cortical “disconnection” as a mechanism of age-related cognitive decline. Neurology.

[39] Sabri O, Ringelstein EB, Hellwig D, Schneider R, Schreckenberger M, Kaiser HJ, Mull M, Buell U (1999). Neuropsychological impairment correlates with hypoperfusion and hypometabolism but not with severity of white matter lesions on MRI in patients with cerebral microangiopathy. Stroke.

[40] O’Sullivan M, Morris RG, Huckstep B, Jones DK, Williams SC, Markus HS (2004). Diffusion tensor MRI correlates with executive dysfunction in patients with ischaemic leukoaraiosis. J Neurol Neurosurg Psychiatry.

[41] Pierpaoli C, Basser PJ (1996). Toward a quantitative assessment of diffusion anisotropy. Magn Reson Med.

[42] Beaulieu C, Allen PS (1994). Water diffusion in the giant axon of the squid: implications for diffusion-weighted MRI of the nervous system. Magn Reson Med.

[43] Jones DK (2008). Studying connections in the living human brain with diffusion MRI. Cortex.

[44] Wheeler-Kingshott CA, Cercignani M (2009). About “axial” and “radial” diffusivities. Magn Reson Med.

[45] Jones DK, Knosche TR, Turner R (2013). White matter integrity, fiber count, and other fallacies: the do’s and don’ts of diffusion MRI. Neuroimage.

[46] Nitkunan A, Barrick TR, Charlton RA, Clark CA, Markus HS (2008). Multimodal MRI in cerebral small vessel disease: its relationship with cognition and sensitivity to change over time. Stroke.

[47] Molko N, Pappata S, Mangin JF, Poupon F, LeBihan D, Bousser MG, Chabriat H (2002). Monitoring disease progression in CADASIL with diffusion magnetic resonance imaging: a study with whole brain histogram analysis. Stroke.

[48] Holtmannspotter M, Peters N, Opherk C, Martin D, Herzog J, Bruckmann H, Samann P, Gschwendtner A, Dichgans M (2005). Diffusion magnetic resonance histograms as a surrogate marker and predictor of disease progression in CADASIL: a two-year follow-up study. Stroke.

[49] O’Sullivan M, Jones DK (2011). Diffusion in chronic stroke and small vessel disease. Diffusion MRI. Theory, methods and applications.

[50] Berlot R, Metzler-Baddeley C, Jones DK, O’Sullivan MJ (2014). CSF contamination contributes to apparent microstructural alterations in mild cognitive impairment. Neuroimage.

[51] O’Sullivan M, Barrick TR, Morris RG, Clark CA, Markus HS (2005). Damage within a network of white matter regions underlies executive dysfunction in CADASIL. Neurology.

[52] van der Holst HM, Tuladhar AM, van Norden AG, de Laat KF, van Uden IW, van Oudheusden LJ, Zwiers MP, Norris DG, Kessels RP, de Leeuw FE (2013). Microstructural integrity of the cingulum is related to verbal memory performance in elderly with cerebral small vessel disease: the RUN DMC study. Neuroimage.

[53] Correia S, Lee SY, Voorn T, Tate DF, Paul RH, Zhang S, Salloway SP, Malloy PF, Laidlaw DH (2008). Quantitative tractography metrics of white matter integrity in diffusion-tensor MRI. Neuroimage.

[54] Lawrence AJ, Chung AW, Morris RG, Markus HS, Barrick TR (2014). Structural network efficiency is associated with cognitive impairment in small-vessel disease. Neurology.

[55] Reijmer YD, Fotiadis P, Martinez-Ramirez S, Salat DH, Schultz A, Shoamanesh A, Ayres AM, Vashkevich A, Rosas D, Schwab K (2015). Structural network alterations and neurological dysfunction in cerebral amyloid angiopathy. Brain.

[56] Sappey Marinier D, Calabrese G, Hetherington HP, Fisher SN, Deicken R, Van Dyke C, Fein G, Weiner MW (1992). Proton magnetic resonance spectroscopy of human brain: applications to normal white matter, chronic infarction, and MRI white matter signal hyperintensities. Magn Reson Med.

[57] Brooks WM, Wesley MH, Kodituwakku PW, Garry PJ, Rosenberg GA (1997). 1H-MRS differentiates white matter hyperintensities in subcortical arteriosclerotic encephalopathy from those in normal elderly. Stroke.

[58] Maillard P, Fletcher E, Lockhart SN, Roach AE, Reed B, Mungas D, DeCarli C, Carmichael OT (2014). White matter hyperintensities and their penumbra lie along a continuum of injury in the aging brain. Stroke.

[59] Taheri S, Gasparovic C, Huisa BN, Adair JC, Edmonds E, Prestopnik J, Grossetete M, Shah NJ, Wills J, Qualls C (2011). Blood-brain barrier permeability abnormalities in vascular cognitive impairment. Stroke.

[60] Huisa BN, Caprihan A, Thompson J, Prestopnik J, Qualls CR, Rosenberg GA (2015). Long-term blood-brain barrier permeability changes in Binswanger disease. Stroke.

[61] Rosenberg GA, Wallin A, Wardlaw JM, Markus HS, Montaner J, Wolfson L, Iadecola C, Zlokovic BV, Joutel A, Dichgans M (2016). Consensus statement for diagnosis of subcortical small vessel disease. J Cereb Blood Flow Metab.

[62] Heiss WD, Zimmermann-Meinzingen S (2012). PET imaging in the differential diagnosis of vascular dementia. J Neurol Sci.

[63] Benson DF, Kuhl DE, Hawkins RA, Phelps ME, Cummings JL, Tsai SY (1983). The fluorodeoxyglucose 18 F scan in Alzheimer’s disease and multi-infarct dementia. Arch Neurol.

[64] Mielke R, Herholz K, Grond M, Kessler J, Heiss WD (1992). Severity of vascular dementia is related to volume of metabolically impaired tissue. Arch Neurol.

[65] Herholz K, Salmon E, Perani D, Baron JC, Holthoff V, Frolich L, Schonknecht P, Ito K, Mielke R, Kalbe E (2002). Discrimination between Alzheimer dementia and controls by automated analysis of multicenter FDG PET. Neuroimage.

[66] Bohnen NI, Djang DS, Herholz K, Anzai Y, Minoshima S (2012). Effectiveness and safety of 18 F-FDG PET in the evaluation of dementia: a review of the recent literature. J Nucl Med.

[67] Su L, Cai Y, Xu Y, Dutt A, Shi S, Bramon E (2014). Cerebral metabolism in major depressive disorder: a voxel-based meta-analysis of positron emission tomography studies. BMC Psychiatry.

[68] Herholz K, Weisenbach S, Kalbe E, Diederich NJ, Heiss WD (2005). Cerebral acetylcholine esterase activity in mild cognitive impairment. Neuroreport.

[69] Hilker R, Thomas AV, Klein JC, Weisenbach S, Kalbe E, Burghaus L, Jacobs AH, Herholz K, Heiss WD (2005). Dementia in Parkinson disease: functional imaging of cholinergic and dopaminergic pathways. Neurology.

[70] Braak H, Braak E (1991). Neuropathological stageing of Alzheimer-related changes. Acta Neuropathol.

[71] Klunk WE, Engler H, Nordberg A, Wang Y, Blomqvist G, Holt DP, Bergstrom M, Savitcheva I, Huang GF, Estrada S (2004). Imaging brain amyloid in Alzheimer’s disease with Pittsburgh Compound-B. Ann Neurol.

[72] Villemagne VL, Mulligan RS, Pejoska S, Ong K, Jones G, O’Keefe G, Chan JG, Young K, Tochon-Danguy H, Masters CL (2012). Comparison of 11C-PiB and 18 F-florbetaben for Abeta imaging in ageing and Alzheimer’s disease. Eur J Nucl Med Mol Imaging.

[73] Aizenstein HJ, Nebes RD, Saxton JA, Price JC, Mathis CA, Tsopelas ND, Ziolko SK, James JA, Snitz BE, Houck PR (2008). Frequent amyloid deposition without significant cognitive impairment among the elderly. Arch Neurol.

[74] Herholz K, Ebmeier K (2011). Clinical amyloid imaging in Alzheimer’s disease. Lancet Neurol.

[75] Yotter RA, Doshi J, Clark V, Sojkova J, Zhou Y, Wong DF, Ferrucci L, Resnick SM, Davatzikos C (2013). Memory decline shows stronger associations with estimated spatial patterns of amyloid deposition progression than total amyloid burden. Neurobiol Aging.

[76] Maruyama M, Shimada H, Suhara T, Shinotoh H, Ji B, Maeda J, Zhang MR, Trojanowski JQ, Lee VM, Ono M (2013). Imaging of tau pathology in a tauopathy mouse model and in Alzheimer patients compared to normal controls. Neuron.

[77] Small GW, Bookheimer SY, Thompson PM, Cole GM, Huang SC, Kepe V, Barrio JR (2008). Current and future uses of neuroimaging for cognitively impaired patients. Lancet Neurol.

[78] Spillantini MG, Goedert M (2013). Tau pathology and neurodegeneration. Lancet Neurol.

[79] Snowdon DA, Greiner LH, Mortimer JA, Riley KP, Greiner PA, Markesbery WR (1997). Brain infarction and the clinical expression of Alzheimer disease. The Nun Study. JAMA.

[80] Pendlebury ST (2012). Dementia in patients hospitalized with stroke: rates, time course, and clinico-pathologic factors. Int J Stroke.

[81] Whitehead SN, Hachinski VC, Cechetto DF (2005). Interaction between a rat model of cerebral ischemia and beta-amyloid toxicity: inflammatory responses. Stroke.

[82] Thiel A, Heiss WD (2011). Imaging of microglia activation in stroke. Stroke.

[83] Stefaniak J, O’Brien J (2016). Imaging of neuroinflammation in dementia: a review. J Neurol Neurosurg Psychiatry.

[84] Schmitt AB, Brook GA, Buss A, Nacimiento W, Noth J, Kreutzberg GW (1998). Dynamics of microglial activation in the spinal cord after cerebral infarction are revealed by expression of MHC class II antigen. Neuropathol Appl Neurobiol.

[85] Thiel A, Radlinska BA, Paquette C, Sidel M, Soucy JP, Schirrmacher R, Minuk J (2010). The temporal dynamics of poststroke neuroinflammation: a longitudinal diffusion tensor imaging-guided PET study with 11C-PK11195 in acute subcortical stroke. J Nucl Med.

[86] Radlinska BA, Blunk Y, Leppert IR, Minuk J, Pike GB, Thiel A (2012). Changes in callosal motor fiber integrity after subcortical stroke of the pyramidal tract. J Cereb Blood Flow Metab.

[87] Thiel A, Cechetto DF, Heiss WD, Hachinski V, Whitehead SN (2014). Amyloid burden, neuroinflammation, and links to cognitive decline after ischemic stroke. Stroke.

